# Spin-state-dependent electrical conductivity in single-walled carbon nanotubes encapsulating spin-crossover molecules

**DOI:** 10.1038/s41467-021-21791-3

**Published:** 2021-03-11

**Authors:** Julia Villalva, Aysegul Develioglu, Nicolas Montenegro-Pohlhammer, Rocío Sánchez-de-Armas, Arturo Gamonal, Eduardo Rial, Mar García-Hernández, Luisa Ruiz-Gonzalez, José Sánchez Costa, Carmen J. Calzado, Emilio M. Pérez, Enrique Burzurí

**Affiliations:** 1 IMDEA Nanociencia, Campus de Cantoblanco, Madrid, Spain; 2 Departamento de Química Física, Universidad de Sevilla, Sevilla, Spain; 3 Materials Science Factory, Instituto de Ciencia de Materiales de Madrid (ICMM), Consejo Superior de Investigaciones Científicas (CSIC), Madrid, Spain; 4 Departamento de Quimica Inorgánica, Universidad Complutense de Madrid, Madrid, Spain

**Keywords:** Electronic devices, Molecular electronics, Electronic properties and materials

## Abstract

Spin crossover (SCO) molecules are promising nanoscale magnetic switches due to their ability to modify their spin state under several stimuli. However, SCO systems face several bottlenecks when downscaling into nanoscale spintronic devices: their instability at the nanoscale, their insulating character and the lack of control when positioning nanocrystals in nanodevices. Here we show the encapsulation of robust Fe-based SCO molecules within the 1D cavities of single-walled carbon nanotubes (SWCNT). We find that the SCO mechanism endures encapsulation and positioning of individual heterostructures in nanoscale transistors. The SCO switch in the guest molecules triggers a large conductance bistability through the host SWCNT. Moreover, the SCO transition shifts to higher temperatures and displays hysteresis cycles, and thus memory effect, not present in crystalline samples. Our results demonstrate how encapsulation in SWCNTs provides the backbone for the readout and positioning of SCO molecules into nanodevices, and can also help to tune their magnetic properties at the nanoscale.

## Abstract (web summary)

Spin-crossover molecules can change their spin-state under a variety of stimuli making them ideal sensors; however, they are typically insulating and unstable. Here, Villalva et al overcome these limitations by encapsulating spin-crossover molecules inside a carbon nanotube.

## Introduction

Magnetic molecules confirm an ever-growing and diverse family of functional units that could add a new twist to conventional electronics provided that their complex properties can be implemented into electronic or spintronic circuits and nanodevices^1^. Spin-crossover (SCO) molecules have lately attracted special interest owing to their ability to act as a spin switch by modifying their spin state under an external stimulus like light, pressure, or temperature^2–4^. The change in the spin, triggered by an electro-structural switch in the molecule, manifests in macroscale variations in their luminescence, magnetism, volume, and electron properties, providing SCO molecules with spin switch, spin memory, or sensing functionalities, among others^2^. The main challenge SCO molecules face for their implementation in nano-electronics devices is their typically insulating nature. A few reports on direct electron transport through crystals and nanoscale rods show conductance values in the range of the nanoamperes^5,6^ or lower. Several groups have explored alternative paths by embedding SCO particles or molecules in a more conductive matrix, for example, by forming macroscopic composites with carbon nanotubes^7^, other conducting moieties^8–10^, organic conducting polymers^11^, or deposited on graphene^12,13^. Some of these macroscopic hybrid materials are, however, rather incompatible with the fabrication processes required to build nanoscale devices.

Single-walled carbon nanotubes (SWCNTs) can act as model conducting backbones that potentially sense and carry the guest SCO state of the molecules, overcoming their insulating nature. In particular, encapsulation of the SCO compounds within the SWCNT cavity seems a rather attractive approach, as the SWCNTs can be robust mechanical shells that protect SCO molecules from the environment and also serve as vessels to controllably place them in nanoscale devices^14–18^. In spite of the potential interest, only a few examples of other magnetic molecules encapsulated in SWCNTs are reported in the literature^19,20^ to the best of our knowledge.

**Fig. 1:**
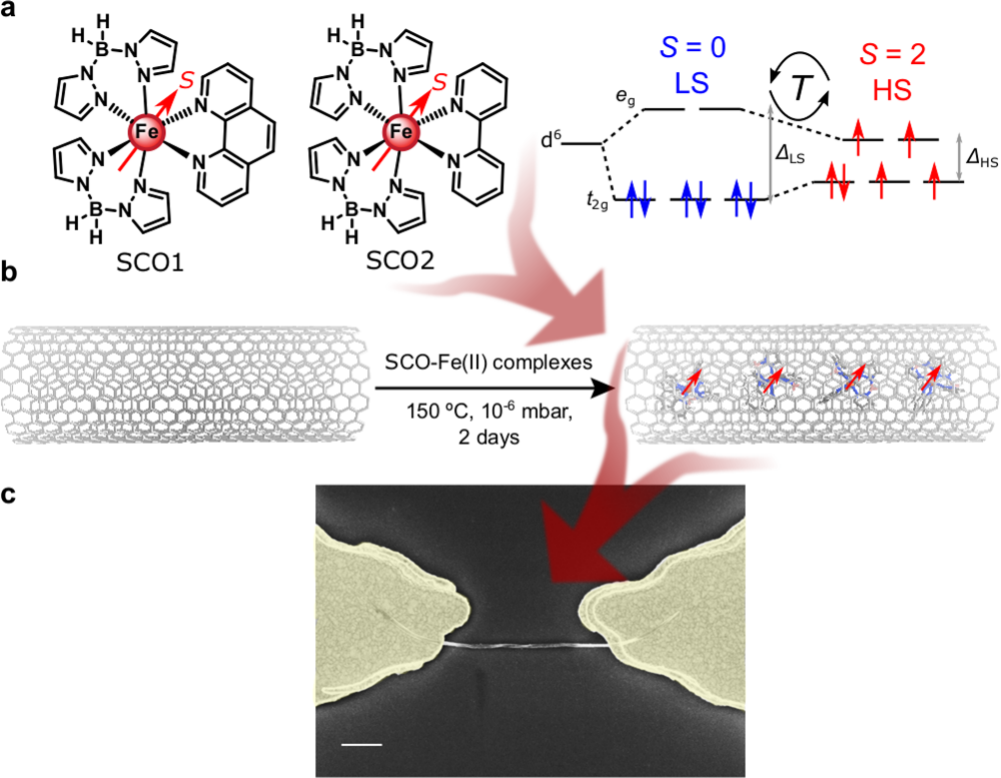
Encapsulation of SCO complexes. **a** Complex SCO1, [Fe(H_2_Bpz_2_)_2_(phen)] and SCO2, [Fe(H_2_Bpz_2_)_2_(bipy)] encapsulated in this work. Spin (*S*) level distribution in the high (HS) and low (LS) spin states of the molecules. **b** Schematic pathway for the encapsulation of Fe (II) SCO complexes in oSWCNTs, resulting in SCO@SWCNT. **c** Scanning electron microscopy (SEM) image of a transistor-like device containing a SCO2@SWCNT heterostructure trapped by dielectrophoresis. Scale bar: 500 nm.

Here, we report the encapsulation of two Fe-based SCO molecules (0D) within SWCNTs (1D) to form mixed-dimensional (0D-1D) SCO@SWCNT hybrids (Fig. 1a, b). We study electron transport through individual SCO@SWCNT heterostructures embedded in nanoscale transistors (Fig. 1c). We show that the host SWCNT conductance is modified by the spin state of the guest encapsulated molecules. The transition between two metastable conductance states is triggered by the thermal SCO switch. In turn, the confinement experienced by the molecules within the SWCNT is translated into a shift of the SCO transition towards higher temperatures and the appearance of a large thermal hysteresis, not present in bulk. We argue that the origin of this bidirectional interaction could be a combination of mechanical and electrostatic effects. The analysis of the experimental results is supported by density functional theory (DFT) calculations.

## Results and discussion

The SCO complexes [Fe(H_2_Bpz_2_)_2_(L)] (H_2_Bpz_2_ = dihydrobis(pyrazolyl)borate, *L* = 1,10-phenanthroline (phen, SCO1), or 2,2’-bipyridine (bipy, SCO2) shown in Fig. 1a were chosen for encapsulation owing to their ability to be sublimed^21,22^ and their SCO robustness when deposited as thin films^23,24^ or even individual molecules^25,26^ on different surfaces. The SCO behavior of these two complexes has already been studied in bulk and thin-film state. They present a thermally induced spin-crossover transition at 167 K (SCO1) and 160 K (SCO2) from a high-spin (HS, *S* = 2) to a low-spin (LS, *S* = 0) state, as evidenced from the magnetization data of the powder samples (see Supplementary Figs. 1 and 2)^26,27^. The SWCNTs used were commercially available chemical vapor deposition (CVD)-grown SWCNTs with diameters ranging from 1.6 to 2.2 nm (length 3–30 μm, 99% purity). In order to encapsulate the complexes, the nanotubes were previously opened by thermal oxidation in the air atmosphere (ca. 40% weight was lost in the process). The metallic impurities were removed by two sequential acid washes, after which the metallic residue was reduced to 4.6%. For the encapsulation, SCO1 or SCO2 and the opened SWCNTs (oSWCNTs) were sealed in a quartz ampoule at 10^−6^ mbar and then heated at 150 °C for 2 and 7 days, respectively. The selected temperature is close to the experimental sublimation temperature found at 10^−2^ mbar (162 °C for SCO1 and 160 °C for SCO2)^21^. The applied low pressures ensure the sublimation of both complexes. The complex adsorbed on the SWCNT surface was eliminated by washing with tetrachloroethane and dichloromethane, applying 3 min sonication between washes. The washes were stopped when the supernatant solution was completely colorless. See additional details in Supplementary Note 2.

A thorough thermogravimetric analysis (TGA) of the initial SCO complexes, oSWCNTs, and encapsulated samples is shown in Supplementary Note 3 and Supplementary Fig. 3. The encapsulation, schematically shown in Fig. 1b, is confirmed by two key features; the decomposition of the protected SCO organic ligands shifts towards higher temperatures (230–250 °C) as compared with the free molecules (200 °C) and the presence of remaining metal particles after burning the samples until 970 °C. This metallic residue corresponds to the iron core of the complexes. Interestingly, the TGA shows that the encapsulation yields are the same for 2 days and 7 days reactions, therefore 2 days are enough processing time to achieve high filling rates (see Supplementary Fig. 4). Atomic force microscope (AFM) and the TGA study of control samples verify that no adsorbed complexes are present in the encapsulated sample (see Supplementary Fig. 5). In addition, when the initial oSWCNTs are washed following the treatment employed for the encapsulated samples, solvent molecules are able to enter the SWCNTs cavity in high yields. This is not observed when the inner cavity is already occupied with the organometallic complexes (see Supplementary Fig. 6 for further details).

Raman measurements at *λ* = 532, 633, and 785 nm excitation wavelengths of both hybrids were taken on the powder samples at room temperature (see Supplementary Figs. 7–9 and Supplementary Table 1). Surprisingly, when exciting at 532 nm, some SCO1 peaks appear, probably owing to a resonant Raman effect. The peaks in the encapsulated species are blue shifted between 5 and 7 cm^−1^ with respect to the pristine complex. In addition, only certain peaks can be observed. These differences could be indicative of the interaction between molecular and SWCNT phononic modes or changes in the environment of the molecules^28,29^. The SWCNTs characteristic Raman bands are also shifted. The radial breathing modes RBMs are blue shifted in both SCO1@SWCNT and SCO2@SWCNT for those tubes excited with 532 and 633 nm, an alteration previously described for several different encapsulations^30,31^. This effect is not so obvious with the 785 nm laser owing to the excitation of wider SWCNTs, less strained upon encapsulation. A small blue shift of the G band is also observed only in the SCO2@SWCNT derivative (See Supplementary Fig. 9). This shift could be associated with a small charge transfer between SCO molecule and SWCNT, strain, or aggregation effects^13,31–33^. A detailed voltammetry study^34^ to disentangle the charge transfer contribution and subtle differences between SCO molecules will be the subject of a further study. Attenuated total reflection infrared (ATR-IR) spectra of the two different complexes, encapsulated hybrids, and oSWCNTs show the presence of some SCO1 bands in the final hybrid, corresponding to the phenanthroline ligand (see Supplementary Fig. 10 for further details).

**Fig. 2:**
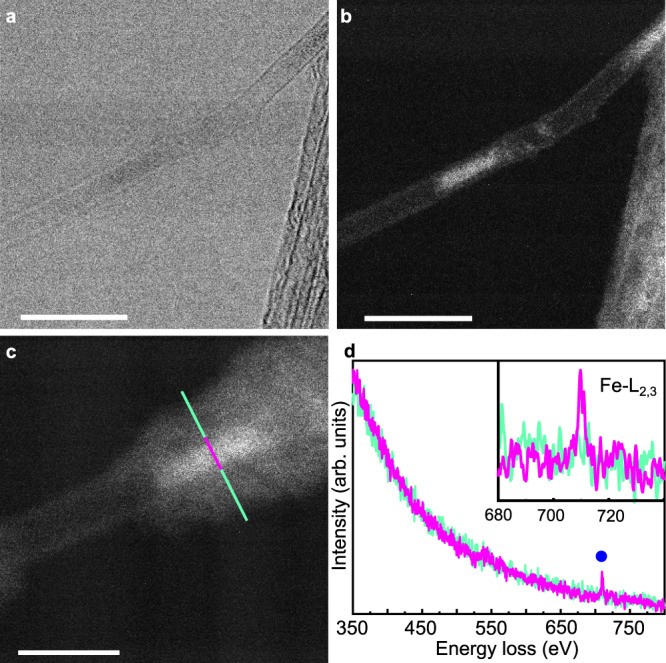
Presence of the Fe-SCO within the SWCNT. **a** Bright-field scanning transmission electron microscope (BF-STEM) image of one SCO1@SWCNT sample (acceleration voltage 80kV). Scale bar: 10 nm. **b** High-angle annular dark-field (HAADF)-STEM image of the SCO1@SWCNT depicted in **a**. Scale bar: 10 nm. **c** Amplified section of **b**. Scale bar: 5 nm. **d** Average electron energy loss spectra (EELS) measured across the turquoise and magenta lines in **c**.

Further evidence for encapsulation is obtained when studying the samples under a scanning transmission electron microscope (STEM). Fig. 2a shows a representative bright-field (BF) STEM image obtained for a SCO1@SWCNT sample. Fig. 2b shows a high-angle annular dark-field (HAADF)-STEM image of a single nanotube with diameter ~1.9 nm. In a HAADF image, the contrast is highly dependent on the atomic number *Z*, being the brightest contrast related to higher atomic number. In this sense, the brighter areas observed inside the tube in Fig. 2b could be related to iron/metal encapsulated atoms with higher *Z* values. The same bright sections appear along the SWCNT bundles, presenting a homogeneous distribution (see Supplementary Fig. 11). Interestingly, the electron energy loss spectra (EELS) taken across one of the bright sections (magenta line in Fig. 2c) clearly shows the signal from the iron L_2,3_ edges appearing, whereas it is absent in the surroundings (turquoise lines), as seen in Fig. 2d. Unequivocally, the encapsulated material correlates with SCO1, as the metal impurities left in the oSWCNT sample are predominantly Mo and Co. The inner structure of the complex cannot be resolved owing to its instability under the electron beam irradiation, which also explains the nanorod-like structure of the sections. In fact, during the EELS analysis, the inner material moves along the nanotube and diverse contaminants adsorb onto the surface of the nanotube—see differences between Fig. 2b and c, which correspond to the same SWCNT section. Note that the analysis of the pristine oSWCNTs under the same conditions reveals no presence of iron (see Supplementary Fig. 12).

Electron transport across individual SCO@SWCNT hybrids is probed in a solid-state field-effect transistor configuration. Sets of 1 μm-spaced gold electrodes are fabricated on a Si/SiO_2_ substrate by mask-less laser lithography and subsequent evaporation of metals. The hybrid SCO@SWCNT derivatives are placed in between the electrodes by dielectrophoresis. This technique is ideally suited to move and position polarized nanomaterials into devices with high precision^17,35,36^. In short, the device is immersed in an isopropanol solution containing the dispersed SCO@SWCNT hybrids. Simultaneously, a sinusoidal AC voltage (frequency *ν* = 3 MHz, amplitude *V* = 3 V, time *t* = 60 s) is applied between the electrodes creating non-homogenous fields. The field-induced SCO@SWCNT dipoles are attracted and aligned into the areas with large gradients, i.e., the gap between the electrodes. The substrate is thereafter blown with nitrogen and annealed during 6 hours at 453 K in vacuum (10^−6^ mbar) to remove excess solvent and organic impurities and to promote the electrical contact between gold and carbon nanotubes. A scanning electron microscopy (SEM) image of a representative device is shown in Fig. 1c. The SEM and AFM imaging and the current-voltage characteristic measured in the device indicate that one or a few SCO@SWCNT hybrids are bridging the gap between the electrodes (See Supplementary Figs. 13 and 17).

**Fig. 3:**
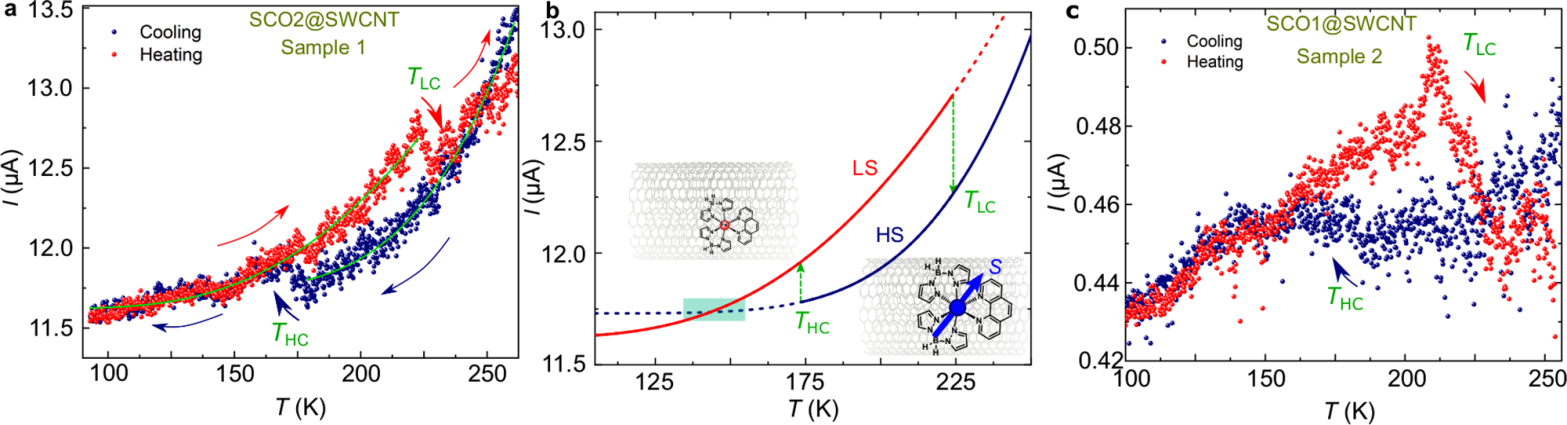
Electron transport across SCO@SWCNT hybrids. **a**, **c** Current *I* measured at a fixed bias *V* = 1 V as a function of the temperature in a SCO2@SWCNT and a SCO1@SWCNT device respectively. The samples are initially cooled down to 90 K (blue curve) and subsequently heated up to room temperature (red curve). The conductance switches between a low-conductance LC and a high-conductance HC state, at temperatures *T*_LC_ and *T*_HC_, respectively, describing a thermal hysteresis. The solid green lines are fit to the HC and LC states with an Arrhenius model for thermally activated transport. **b** Theoretical high-spin HS and low-spin LS curves calculated with a thermally activated transport model (Eq. (1)) and the parameters obtained for sample 1. The HS to LS transition may not be visible in the measurement if *T*_HC_ lies in a temperature range roughly defined by the green square, as seen in some examples in Supplementary Fig. 14.

Figure 3a shows the current/measured at a fixed bias *V* = 1 V as a function of the temperature *T* (5 K/min) in a SCO2@SWCNT device. The sample is initially cooled from room temperature down to 90 K (blue curve) and subsequently heated up back to room temperature (red curve). See Supplementary Fig. 14 for the full measurement. At first, the current decreases monotonically with decreasing temperature. This is indicative of thermally activated transport in semiconducting carbon nanotubes. Interestingly, *I* switches to a high-conductance HC state at around *T*_HC_ = 175 K, close to the SCO transition temperature observed in the crystals of the base molecules^21^. By heating the hybrid back to room temperature, the current remains in the HC state describing a large hysteresis loop with Δ*T* = 50 K and Δ*I* = 0.3 μA until it drops back to the low-conductance LC state in the cooling curve at *T*_LC_ = 223 K. A similar conductance hysteresis loop has been observed at roughly the same temperatures for the other SCO complex in a SCO1@SWCNT device as seen in Fig. 3c. In this case, the LC to HC switch is less clear, whereas the opposite HC to LC switch is more abrupt.

Comparable conductance hysteresis loops are observed in 11 out of 31 (35%) measured devices. See Supplementary Fig. 14 for four additional samples and Supplementary Fig. 15 for a discussion about the stability of the hysteresis in different thermal cycles. No significant statistical differences are observed between SCO1@SWCNT and SCO2@SWCNT in terms of transition temperatures or frequency of the hysteresis in the measurements. In contrast, the switches and thermal hysteresis are not observed in devices made of purified empty carbon nanotubes nor in control samples where the SCO molecules are deposited in supra position onto closed carbon nanotubes and thereafter washed and annealed as in the encapsulated derivatives (see Supplementary Fig. 16 for an example). We therefore ascribe the conductance switch and thermal hysteresis to the SWCNT host sensing the spin switch and spin-state of the guest encapsulated SCO molecules. The HS (LS) state in the molecules would be associated with a lower(higher) conductance metastable states in the hybrid, as seen in the schematics in Fig. 3b. This trend between spin-state and conductance is in agreement with previous reports on electron transport measurements directly across SCO crystals^5^ or nanorods^6,37^ and thin films of SCO nanoparticles/carbon nanotubes composites^7^. In turn, the opposite dependence is observed in SCO monolayers deposited on graphene field-effect transistors^12^ and individual molecules^38,39^. Interestingly, the SCO dynamics can be studied in the transitional regions between conductance (or spin) states, i.e., the width of the LC → HC and the HC → LC transitions. In sample 1, the switch occurs in temperature intervals of ~6 K as in the molecular crystals^21^, indicating that the cooperative effect may be similar. In sample 2, the transition is slightly wider (~25K) as observed in few monolayers with lower cooperativity^40^.

The LS and HS curves can be fitted with an Arrhenius law for thermally activated electron transport:

1 $$I={I}_{0}\exp (-U/{k}_{{\rm{B}}}T)+{I}_{{\rm{i}}}$$

where *I*_0_ is the pre-exponential factor, *U* is the activation barrier, *k*_B_ is the Boltzmann constant and *I*_i_ accounts for temperature-independent contributions to the current. The green solid lines in Fig. 3a are fitting curves with parameters *I*_0_(LS) = (58 ± 4) μA, *U*(LS) = 891 ± 15 K, *I*_i_(LS) = 11.62 ± 0.01 μA, and *I*_0_(HS) = (1770 ± 29) μA, *U*(HS) = 1816 ± 45 K, *I*_i_(HS) = (11.73 ± 0.01) μA for the LS and HS states respectively. The HS to LS switch is therefore associated with a sharp decrease of *I*_0_ and *U* and vice versa. Fig. 3b shows the *IvsT* curves calculated by using Eq. (1). The HS and LS curves of sample 1 can be qualitatively well reproduced by the model. In addition, the model shows how the HS to LS switch may be smaller (as in sample 2) or not be visible (sample 6 in Supplementary Fig. 14), depending on a subtle competition between the transition temperature *T*_HC_ and the thermally activated transport parameters, as explained in refs. ^5,37^ and observed in some examples in the Supplementary Fig. 14.

A striking difference with the magnetic behavior of macroscopic SCO crystalline samples is the appearance of a large thermal hysteresis accompanied by a shift of the SCO transition (especially LS to HS) to higher temperatures. The *T*_LC_ transition is on average 53 K higher than in the bulk compound. Interestingly, a similar though weaker opening of the hysteresis has been reported on SCO nanoparticles when downscaled to the nm scale^41^ and very recently on thin films of our same complexes sublimated onto different surfaces^24^. The thinner the film the wider the hysteresis loop becomes. This tuning of the hysteresis width is explained in terms of the molecule–substrate interaction that significantly modifies the enthalpy difference of the spin states. In particular by stabilizing the LS state as observed in our experiments. In addition, a widening of the hysteresis has been also reported in transport across rod-like SCO crystals^6^. In this different case, the hysteresis is explained in terms of an increasingly high surface-to-volume ratio in cylindrical crystals, however, the mechanism remains unclear. Note that the carbon nanotube can act as template to form rod-like elongated SCO aggregates in its interior, as suggested by the TEM images (see Fig. 2). However, the high *I* values measured in most devices, in the microampere range, allows to safely neglect direct electron transport *via* the typically insulating SCO molecules^5^.

The mechanism behind the SCO-induced switch in the SWCNT conductance, the thermal hysteresis, and its shift to higher temperatures could be of magnetic, mechanical, and/or electrostatic origin. The switch from LS (*S* = 0) to HS (*S* = 2) would effectively trigger a Lorentz force that would introduce an additional source of scattering for the charge carriers in the SWCNT. This would explain the lower conductance of the HS state, but would not by itself explain the appearance of a hysteresis and the shift to higher temperatures. The alternative mechanical and electrostatic mechanisms have been analyzed by DFT calculations.

**Fig. 4:**
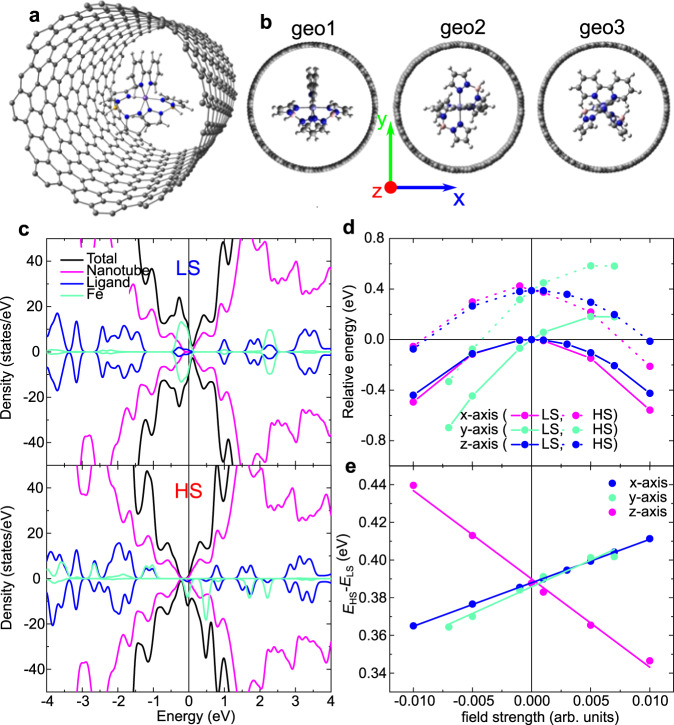
DFT modeling of SCO@SWCNT. **a** Most stable geometry of SCO2 encapsulated on an SWCNT(16,8). **b** Three orientations of the SCO2 molecule with respect to the nanotube wall. **c** Density of states (black lines) and projected density of states on Fe (turquoise), complex ligands (blue) and carbon nanotube (magenta) of the SCO2@SWCNT hybrid for the LS (top) and HS (bottom) states of SCO2. **d** Relative energy (in eV) of the LS (solid lines) and HS (dotted lines) of the SCO2 complex in the presence of an external electric field orientated along the *x*, *y*, or *z* axis. **e** Impact of the applied electric field on the HS–LS energy difference (*E*_HS_−*E*_LS_) of the SCO2 complex.

DFT calculations provide information about the geometries adopted by the SCO molecule once encapsulated and the corresponding interaction energies for HS and LS states, with and without electrical field. More computational details can be found in Supplementary Note 15. The geometry optimization of the SCO2@SWCNT, for both the LS and HS states, has been restrained to three orientations of the bipy group with respect to the nanotube wall. Thus bipy group is placed on the yz, xz, or xy plane, being z the nanotube axis (geo1, geo2, and geo3, respectively, Fig. 4b). The relative energy of the LS and HS states of the encapsulated SCO2 complex are collected in Table 1.

**Table 1:** SCO energy parameters for different geometries.

	Relative energy			Interaction energy	
	(eV)			(eV)	
	LS	HS	HS–LS	LS	HS
geo1	0.000	0.487	0.487	−0.127	−0.080
geo2	0.046	0.519	0.473	−0.081	−0.049
geo3	0.123	0.654	0.530	−0.003	0.086
geo1_1contact	0.285	0.649	0.408	0.159	0.126
geo1_2contacts	0.256	0.725	0.470	0.129	0.158
Isolated molecule			0.440		

The relative energy of the LS and HS spin states and interaction energy for SCO2 complex encapsulated in an SWCNT(16,8), resulting from rPBE periodic calculations at 0 K.

For both spin states, the most stable orientation corresponds to the bipy group placed on the yz plane (geo1, Fig. 4a), but very close to geo2. Two additional sets of calculations have been performed, where the molecule oriented as in geo1 is slightly shifted toward the wall. The displacement can be symmetric, along the *y* axis, introducing a short H....C contact between the bipy group and the wall (geo1_1contact) or along the xy bisector, introducing a second H....C contact between one of the pz groups and the wall (geo1_2contacts). The geometries with short contacts between the molecule and the nanotube show a significant energy penalty with respect to those where the molecule is centered on the nanotube axis. Interestingly, in all cases, the interaction with the nanotube produces a non-negligible enhancement of the LS → HS transition energy (~10–20%). At the same time, the interaction (adsorption) with the nanotube is stronger for the LS than the HS state in all the explored geometries (Table 1). Similar results have been obtained for the [Fe((3,5-(CH_3_)_2_Pz)_3_BH)_2_] complex deposited on the Au(111) surface^42^ as well as for [Fe(phen)_2_(NCS)_2_] on metallic substrates^43,44^. This behavior can be rationalized on the basis of the orbital mixing between the molecule and the nanotube, stronger for the LS state, as shown by the projected density of states in Fig. 4c. In fact, the occupied states close to the Fermi level are centered on the Fe 3d (turquoise curves) with a larger orbital mixing with the ligands (blue curves) for the LS state than for the HS one. These states are then spatially more extended in the case of the LS state, with a non-negligible orbital mixing with the top of the valence band of the nanotube (magenta curves), favoring a stronger molecule-SWCNT interaction for the LS state, and the enhancement of the HS–LS transition energy. This scenario could explain the translation of the spin switch into a distinct bistable conductance in the host nanotube, as well as the shift of the LS–HS transition to higher temperatures (*T*_LC_) observed experimentally. The relevance of the electron orbital mixing with the surface has been suggested also for thin films^24^. The spin-state-dependent molecule-SWCNT mixing could also modify the phonon scattering and therefore the current in the SWCNT, as proposed for graphene^12^.

The impact of the external electric field on the relative energies of the LS and HS states has also been explored by DFT. This is pertinent since the conductance has been measured at a fixed bias voltage and a SCO transition involves a change in the charge distribution of the molecules and thus the dipolar moment^13,45^. We have imposed an external electric field along the *x*, *y*, or z axis, for a single molecule on the center of the nanotube, with the same orientation as in geo1. The applied field perturbs the energy of each spin state on different extension depending on the direction and strength of the field, as seen in Fig. 4d. The HS–LS transition energy (*E*_HS_–*E*_LS_) is significantly enhanced when the field is applied along the nanotube axis (Fig. 4e), which indeed corresponds to the optimal orientation of the encapsulated molecule. This additional mechanism explains and reinforces the increment of the transition temperature observed in our experiments. It could also be behind previous experimental reports on SCO crystals. They show how strong electrical fields, like those present in our devices (10 kV/cm), tend to stabilize the LS state over the HS state^46^ in crystals. The proposed mechanism is the minimization of the potential energy in a larger LS dipole in the presence of electrical fields. This would shift the LS → HS transition to higher temperatures compared with the HS → LS transition, as predicted in our calculations (Fig. 4e), thus opening a thermal hysteresis in the conductance, as observed in our measurements. The electrostatic variation in the SWCNT filling environment could be coupled to the electrical transport via a chemo-electric gating effect that would shift the Fermi level^13^ or as a perturbation of the interfacial phonon modes in the SWCNT as suggested for graphene^12^.

In conclusion, SCO molecules have been encapsulated within the 1D cavities of SWCNTs. The resulting mixed-dimensional hybrids are deterministically positioned in nanoscale field-effect transistors by dielectrophoresis. We show that the SCO mechanism is preserved under encapsulation. The SCO switch, thermally induced in the guest molecule, triggers distinct large-conductance bistability through the host SWCNT. Moreover, the SCO/SWCNT interaction modulates the SCO properties of the guest molecules by shifting the SCO transition to higher temperatures and opening a large hysteresis not present in crystalline samples. Our results demonstrate how encapsulation in SWCNTs provides the mechanical and conducting backbone for the readout and positioning of SCO molecules into nanodevices. In addition, encapsulation in SWCNT can tune the initial properties of the molecules, adding functions like memory effect closer to room temperature. We anticipate that the encapsulation of magnetic molecules in SWCNTs has the potential to become a simple, robust, and high-conducting platform to integrate magnetic molecules in real devices, contributing to surpass the previously reported limitations in the spintronic arena. The 1D nature of SWCNT will allow the formation and study of nanoscale 1D magnetic systems.

## Methods

### Chemicals and reagents

The SWCNTs used were commercially available CVD-grown SWCNTs with diameters ranging from 1.6 to 2.2 nm (length 3–30 μm, 99% purity). The supplier was Cheap Tubes (www.cheaptubes.com). Reagents were used as purchased.

### Thermogravimetric analysis

TGA were performed using a TA Instruments TGAQ500 with a ramp of 10 °C/min under air or nitrogen from 50 to 1000 °C.

### Raman spectroscopy

The powder samples were measured without further treatment. Their Raman spectra were recorded with a Bruker Senterra confocal Raman microscope (Bruker Optic, Ettlingen, Germany, resolution 3–5 cm^−1^) using the following parameters: objective NA 0.75, 50 V; laser excitation: 532 nm: 2 mW, 633 nm: 5 mW, and 785 nm: 10 mW. The samples were deposited on a glass slide.

### Fourier-transform-ATR-IR

ATR-IR was performed with a Bruker ALPHA FT-IR spectrometer.

### Atomic force microscopy

AFM images were acquired using a JPK NanoWizard II AFM working in dynamic mode. NT-MDT NSG01 silicon cantilevers, with typical values of 5.1 N ⋅ m^−1^ spring constant and 150 kHz resonant frequency were employed under ambient conditions in the air.

### Scanning transmission electron microscopy

STEM images were obtained with a JEM ARM 200cF instrument operating at 80 kV (0.08 nm resolution when operating in STEM mode). The samples were dispersed in iPrOH and dropcasted onto 200 square mesh covered by holey carbon.

### Magnetic susceptibility measurements

Magnetic susceptibility measurements between 10 K and 380 K were carried out in a Quantum Design MPMS-5S SQUID magnetometer under a 2000 Oe field. Each sample was secured inside a plastic capsule with cotton. Pascal constants were used to correct for the diamagnetic contribution.

### Electron transport measurements

Temperature-dependent electron transport measurements were performed in a Linkam T95 electrical probe station under nitrogen atmosphere, connected to a Keithley 2450 Sourcemeter.

## Supplementary information

Supplementary Information

## Data Availability

The data that support the findings of this study are available from the corresponding author upon reasonable request. The X-ray crystallographic coordinates for structures reported in this study are deposited at the Cambridge Crystallographic Data Center (CCDC), under deposition numbers 233281 and 233285. These data can be obtained free of charge from The Cambridge Crystallographic Data Center via www.ccdc.cam.ac.uk/data_request/cif.

## Code availability

The periodic DFT calculations are carried out with Vienna Ab initio Simulation Package code, a copyright-protected software, distributed by VASP Software GmbH (https://www.vasp.at). The simulation of the impact of the external electric field on the HS–LS energy gap is performed with Gaussian 09 code, distributed by Gaussian, Inc (https://www.gaussian.com). Chemcraft code was employed to prepare the images of the encapsulated molecules of Fig. 4. https://www.chemcraftprog.com/
