# Supplementary material for: Spin-state-dependent electrical conductivity in single-walled carbon nanotubes encapsulating spin-crossover molecules
Source: Nat Commun. 2021 Mar 11;12:1578. doi: 10.1038/s41467-021-21791-3 (PMC7952721; doi:10.1038/s41467-021-21791-3)
Supplement: Supplementary file 1 — Supplementary Information [file 41467_2021_21791_MOESM1_ESM.pdf]

Supplementary Information

Spin-state dependent electrical conductivity in single-wall carbon nanotubes encapsulating spin crossover molecules

Julia Villalva[1], Aysegul Develioglu[1], Nicolas Montenegro-Pohlhammer[2], Rocío Sánchez-de-Armas[2], Arturo Gamonal[1], Eduardo Rial[1], Mar García-Hernández[3], Luisa Ruiz-Gonzalez[4], José Sánchez Costa[1], Carmen J. Calzado[2], Emilio M. Pérez[1] and Enrique Burzurí[1]

[1]IMDEA Nanociencia, Campus de Cantoblanco, Calle Faraday 9, 28049 Madrid, Spain
[2]Departamento de Química Física, Universidad de Sevilla, c/Profesor García González, s/n 41012 Sevilla, Spain
[3]Materials Science Factory, Instituto de Ciencia de Materiales de Madrid (ICMM), Consejo Superior de Investigaciones Científicas (CSIC), Sor Juana Inés de la Cruz 3, 28049 Madrid, Spain
[4]Departamento de Química Inorgánica, Universidad Complutense de Madrid, 28040 Madrid, Spain

INDEX

Supplementary Note 1. Synthesis of SCO1 and SCO2 2
Supplementary Note 2. Detailed procedure for the encapsulation of SCO in SWCNTs 3
Supplementary Note 3. TGA results for the encapsulation of SCO1 and SCO2 4
Supplementary Note 4. Encapsulation yields in SCO1@SWCNTs for 2- and 7-days reactions 5
Supplementary Note 5. Supramolecular controls 6
Supplementary Note 6. TGA Control Samples 7
Supplementary Note 7. Raman spectra for 633 nm laser excitation 7
Supplementary Note 8. Raman spectra for 532 and 785 nm laser excitation 8
Supplementary Note 9. Raman displacements 8
Supplementary Note 10. 2D vs G plots in 532 and 633 nm lasers 9
Supplementary Note 11. ATR-IR spectra 9
Supplementary Note 12. HAADF-STEM of a bundle of SCO1@SWCNTs 10
Supplementary Note 13. HAADF-STEM and EELS of pristine open SWCNTs 10
Supplementary Note 14. Additional electron transport measurements and AFM images of devices 11
Supplementary Note 15. Computational details 14
Supplementary References 15

Supplementary Note 1. Synthesis of SCO1 and SCO2

[Fe(H_2Bpz$_2$)$_2$phen], (SCO1). SCO1 was synthesized following the procedure previously described.[1]

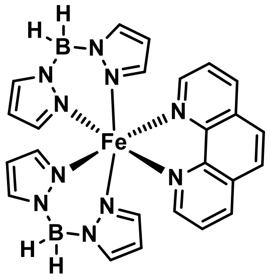

$Fe(BF_4)_2 \cdot 6H_2O$ (0.46 g, 1.4 mmol) was added to a potassium bis(1-pyrazolyl)borohydrate (0.50 g, 2.7 mmol) methanolic solution (13 mL) with ascorbic acid (5 mg) and stirred for 1 h under argon atmosphere. The resulting KBF_4 salt was eliminated by filtration and phenanthroline (0.27 g, 1.4 mmol) was added under stirring to the filtrate forming a dark violet precipitate. The violet microcrystalline powder was filtered, washed twice using methanol (10 mL) and dried under vacuum (0.42 g, 65%). IR spectroscopy, XRD and magnetic characterization match the data described in literature.

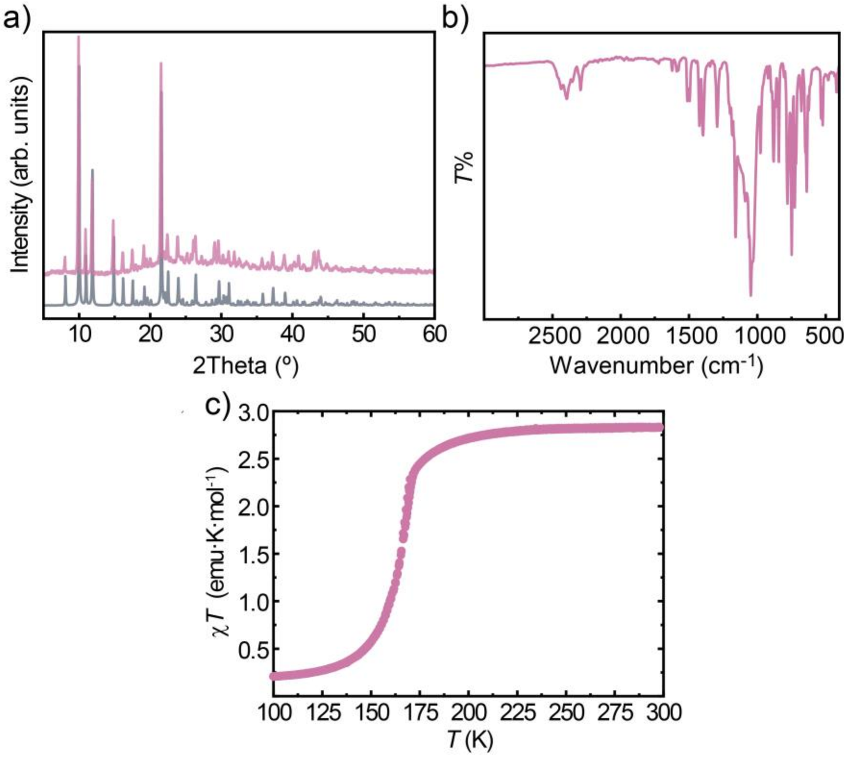

***Supplementary Figure 1*.The SCO1 structure and magnetism.** Experimental XRD pattern of SCO1 (a, pink) and calculated pattern obtained from single crystal structure (a, grey). Crystallographic data was obtained from Real *et al.*[1] b) IR spectrum obtained for SCO1. c*)* χT *vs* T curve of SCO1, $T_{1/2}$ = 167 K.

[Fe(H_2Bpz$_2$)$_2$bipy], (SCO2). SCO2 was synthesized following the procedure previously described.

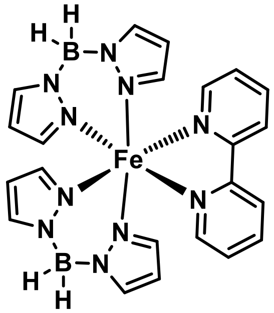

$Fe(BF_4)_2 \cdot 6H_2O$ (0.46 g, 1.4 mmol) was added to a potassium bis(1-pyrazolyl)borohydrate (0.50 g, 2.7 mmol) methanolic solution (13 mL) with ascorbic acid (5 mg) and stirred for 1 h under argon atmosphere. The resulting KBF_4 salt was eliminated by filtration and 2,2'-bipyridine (0.22 g, 1.4 mmol) was added under stirring to the filtrate forming a dark violet precipitate. The violet microcrystalline powder was filtered, washed twice using methanol (10 mL) and dried under vacuum (0.37 g, 52%). IR spectroscopy, XRD and magnetic characterization match the data described in literature.

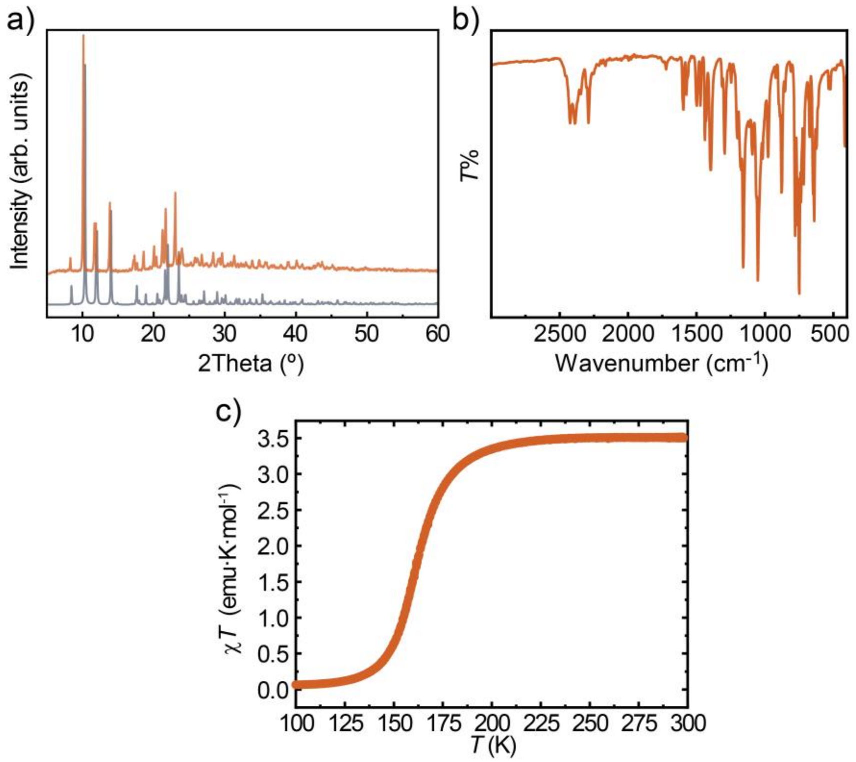

***Supplementary Figure 2*. The SCO2 structure and magnetism.** Experimental XRD pattern of SCO2 (a, orange) and calculated pattern obtained from single crystal structure (a, grey). Crystallographic data was obtained from Real, et al [1] b) IR spectrum obtained for SCO2. c) χT *vs* T curve of SCO2, $T_{1/2}$ = 160 K.

Supplementary Note 2. Detailed procedure for the encapsulation of SCO in SWCNTs

1.- SWCNT pre-treatment

100 mg SWCNTs purchased from Cheap Tubes, Inc. were previously opened by thermal oxidation in air atmosphere at 600 ºC for 45 min (ca. 40% weight was lost in the process). They were then purified by two sequential acid washes: the nanotubes were suspended in 40 mL HCl 35% (1.5 mg SWCNT/mL HCl) and sonicated for 10 min. The mixture was poured in 120 mL miliQ water and filtered through a 0.2 µm pore-sized polycarbonate membrane. The solid obtained was washed with water until neutral pH and then dried in an oven at 150 ºC for 90 min. The cleaning procedure was repeated twice, and the metallic residue was reduced to 4.6% (according to TGA).

2.-Procedure for the encapsulation

For the encapsulation, 90 mg SCO1 or SCO2 and 25 mg opened SWCNTs (oSWCNTs) were sealed in a quartz ampoule at 10^{-6} mbar and then heated in an oven at 150 ºC for 2 and 7 days. The selected temperature is close to the experimental sublimation temperature found at 10^{-2} mbar (162 ºC for SCO1 and 160 ºC for SCO2). The applied low pressures ensure the sublimation of both complexes. The complex adsorbed on the SWCNT surface was eliminated by washing with tetrachloroethane and dichloromethane, applying 3 min sonication between washes. The washes were stopped when the supernatant solution was completely colourless.

Supplementary Note 3. TGA results for the encapsulation of SCO1 and SCO2

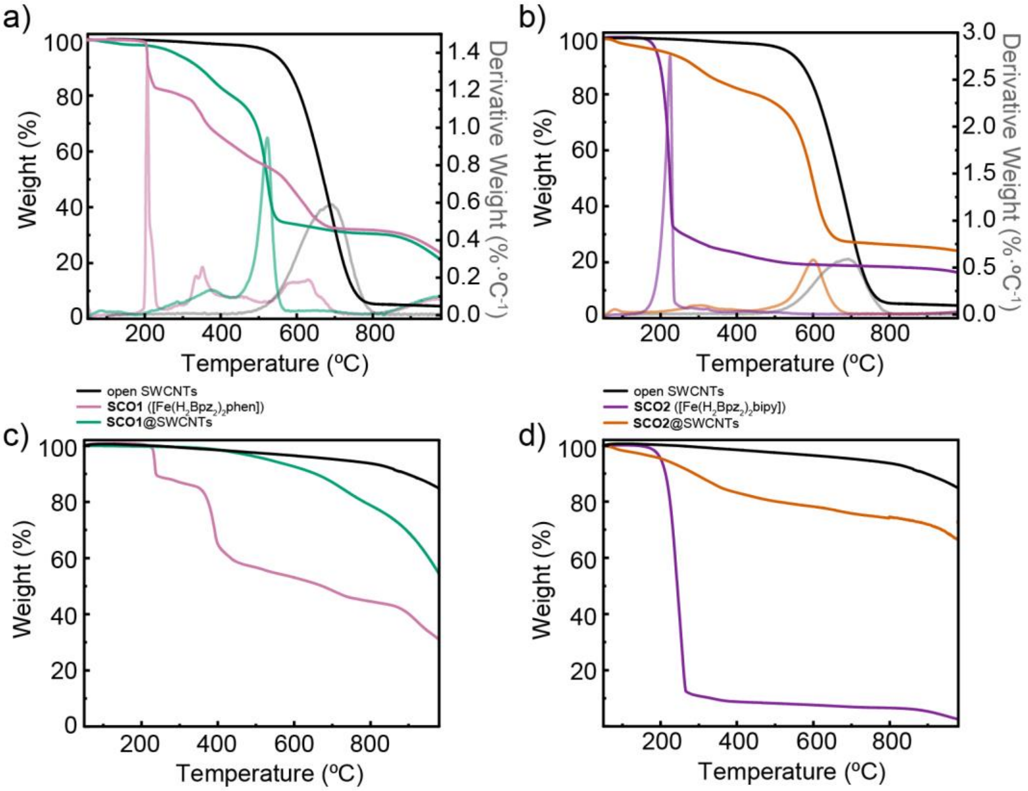

***Supplementary Figure 3*.TGA for encapsulation of SCO1 and SCO2.** a) TGA analysis (Air, 10 ºC·min^{-1}) of open SWCNT (black), SCO1@SWCNTs (green) and SCO1 (pink), b) TGA analysis (Air, 10 ºC·min^{-1}) of open SWCNT (black), SCO2@SWCNTs (orange) and SCO2 (purple). c) and d) TGA analysis (N_2, 10 ºC·min^{-1}) of the same samples, colour reference is maintained.

The results obtained for the TGA analysis are similar for both encapsulations, as expected given the structural similarity of the complexes. For SCO1@SWCNTs, the plot obtained in air shows a first weight loss that starts at around 250 ºC and ends up combined with the SWCNTs loss. This loss has been adscribed as the de-encapsulation and burning of the organic ligands. This curve differs from that of pure SCO1, with has three different losses, the first one starting at 200 ºC. In the case of SCO2@SWCNTs, the ligands weight loss is situated at 230 ºC, while SCO2 decomposes almost completely at 160 ºC. This shifts towards higher temperatures are explained by the protective shield that constitutes the SWCNTs wall. The key feature in both cases is the remaining metal particles after burning the sample until 970 ºC., probably already in its oxidized state. In the nitrogen spectra there is as well a displacement between the encapsulated weight losses and pristine complexes.

Supplementary Note 4. Encapsulation yields in SCO1@SWCNTs for 2- and 7-days reactions

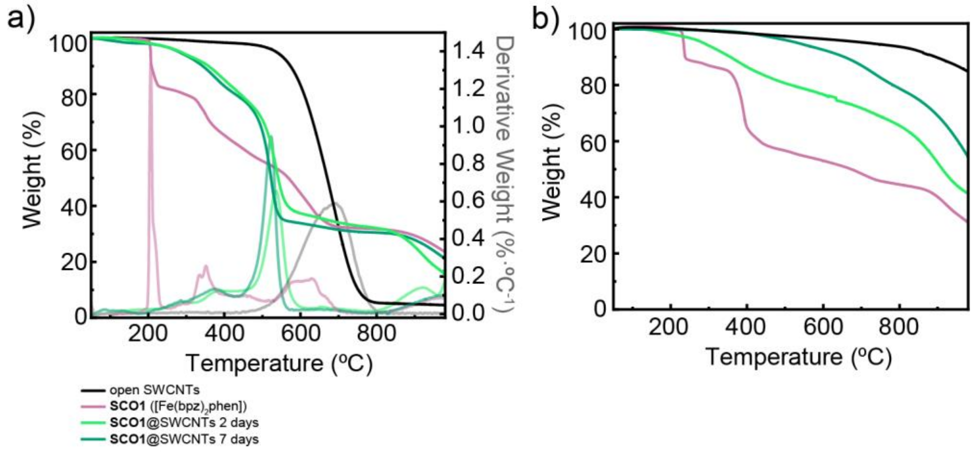

Supplementary Figure 4. **Encapsulation yields in SCO1@SWCNTs.** a) TGA analysis (Air, 10 ºC·min^{-1}) of open SWCNT (black), **SCO1**@SWCNTs after 2 days reaction (pale green), SCO1@SWCNTs after 7 days reaction (dark green) and SCO1 (pink). b) TGA analysis (N_2, 10 ºC·min^{-1}) of the same.

The differences observed when N_2 is employed can be caused by a slight presence of oxygen in the TGA chamber.

Supplementary Note 5. Supramolecular controls

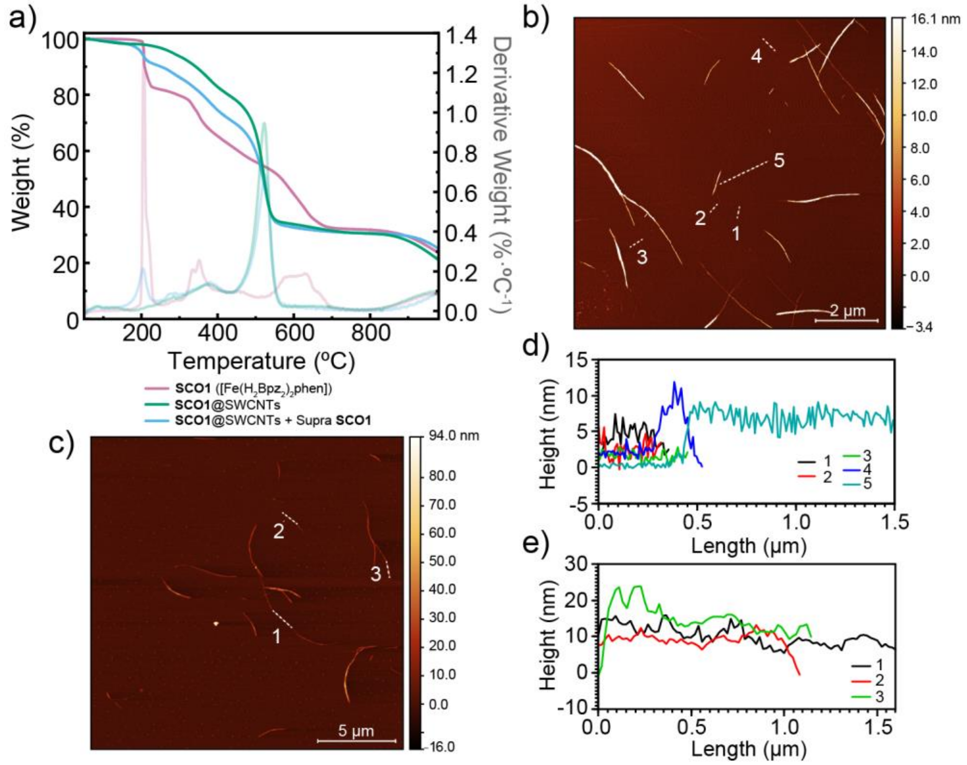

Supplementary Figure 5**.Supramolecular controls.** TGA analysis (Air, 10 ºC min^{-1}) of Open SWCNT (black), SCO1@SWCNTs (green), SCO1 (pink) and control sample SCO1-SCO1@oSWCNTs (blue). b) AFM micrograph of the pristine open SWCNTs dropcasted and dried over a freshly exfoliated mica substrate. c) AFM micrograph of SCO1@SWCNTs sample dropcasted and dried over a freshly exfoliated mica substrate. d) Height profiles of five different longitudinal sections selected in b). e) Height profiles of three different longitudinal sections selected in c).

Supplementary Figure **5**a shows a supramolecular SCO1-SCO1@SWCNTs thermogravimetric analysis. The supramolecular hybrid presents a new loss at almost the same temperature as pristine SCO1, confirming that no adsorbed complexes are present in the encapsulated sample. This is also verified under AFM (Supplementary Supplementary Figure **5**b-e), where the height profile along the pristine open SWCNTs and filled open SWCNTs follow the same patterns, *i.e.* there is no increase in rugosity due to physisorbed complexes.

Supplementary Note 6. TGA Control Samples

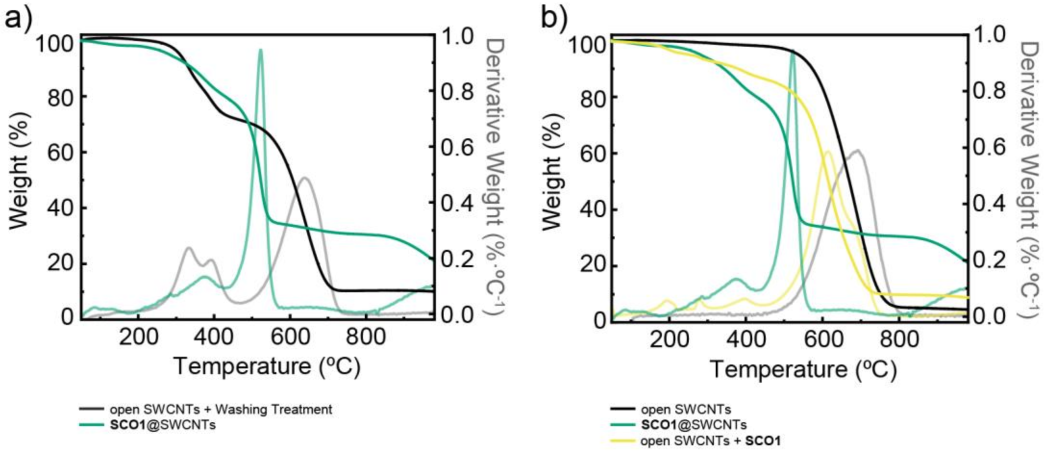

Supplementary Figure 6. **TGA control samples.** a) TGA analysis (Air, 10 ºC·min^{-1}) of open SWCNT with washing treatment (black) and SCO1@SWCNTs (green). b) TGA analysis (Air, 10 ºC·min^{-1}) of open SWCNT (black), SCO1@SWCNTs (green) and SCO1-SWCNTs (yellow).

Two different control samples were prepared. The first one, Supplementary Figure 6a, shows the differences between the pristine open SWCNTs after repeating the necessary washes to remove adsorbed compound SCO1 and the encapsulated sample. In Supplementary Figure 6b, the previously analyzed samples are plotted with the supramolecular hybrid SCO1-SWCNTs, which presents different weight losses than SCO1@SWCNTs.

Supplementary Note 7. Raman spectra for 633 nm laser excitation

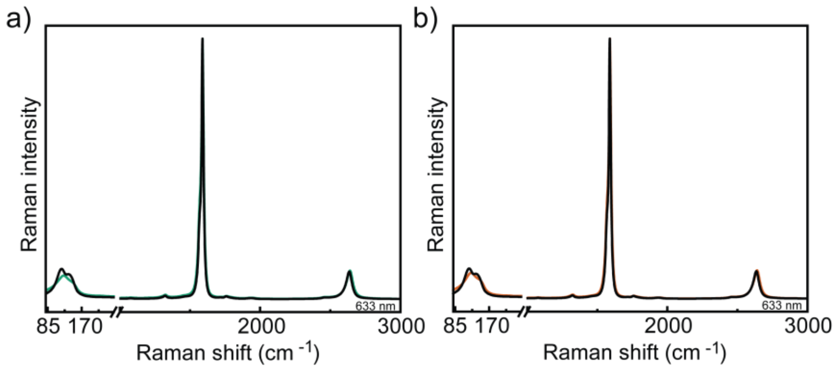

Supplementary Figure 7**.** **Raman spectra for 633 nm excitation.** a) Raman spectra of open SWCNTs (black line) and SCO1@SWCNTs (green line) for 633 nm laser. b) Raman spectra of open SWCNTs (black line) and SCO2@SWCNTs (orange line) for 633 nm laser.

Supplementary Note 8. Raman spectra for 532 and 785 nm laser excitation

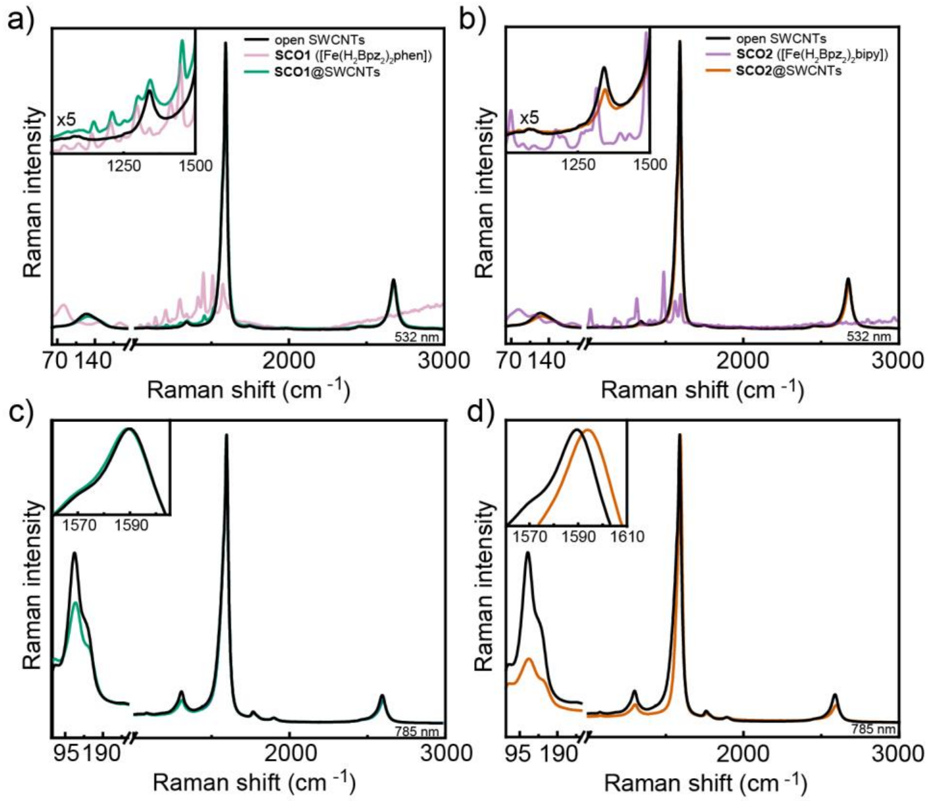

***Supplementary Figure 8*. Raman spectra for 532 and 785 excitation**. Raman spectra of open SWCNTs (black), SCO1@SWCNTs (green) and SCO1 (pink) for a) 532 nm laser and c) 785 nm laser. Raman spectra of open SWCNTs (black), SCO2@SWCNTs (orange) and SCO2 (purple) for b) 532 nm laser and d) 785 nm laser. The Raman spectra shown are the average of around 100 different spectra.

Supplementary Note 9. Raman displacements

532 nm				
	RBM (cm^{-1})	D (cm^{-1})	G (cm^{-1})	2D (cm^{-1})
SCO1@SWCNTs	127 ± 2	1343 ± 2	1592 ± 1	2677 ± 1
SCO2@SWCNTs	125 ± 2	1343 ± 1	1594 ± 1	2678 ± 2
SWCNTs	120 ± 2	1338 ± 1	1591 ± 1	2671 ± 1
633 nm				
	RBM (cm^{-1})	D (cm^{-1})	G (cm^{-1})	2D (cm^{-1})
SCO1@SWCNTs	126 ± 1	1325 ± 1	1592 ± 1	2642 ± 1
SCO2@SWCNTs	121 ± 1	1323 ± 2	1595 ± 1	2639 ± 1
SWCNTs	120 ± 1	1322 ± 1	1591 ± 1	2635 ± 2
785 nm				
	RBM (cm^{-1})	D (cm^{-1})	G (cm^{-1})	2D (cm^{-1})
SCO1@SWCNTs	118 ± 2	1303 ± 2	1589 ± 1	2594 ± 2
SCO2@SWCNTs	119 ± 2	1303 ± 2	1595 ± 1	2597 ± 2
SWCNTs	115 ± 1	1300 ± 1	1589 ± 1	2588 ± 2

***Supplementary Table 1.* Average Raman displacements.** Average displacements from 100 different Raman spectra with their correspondent standard deviation. The Radial Breathing Modes, D band, G band and 2D band are presented. The Raman spectra were recorded using the powder samples deposited on a glass surface. When the SWCNTs are excited with 785 nm, a displacement in the G band is observed. A student's t-test confirms that samples SCO1@SWCNTs and SCO2@SWCNTs are different from pristine SWCNTs.

Supplementary Note 10. 2D vs G plots in 532 and 633 nm lasers

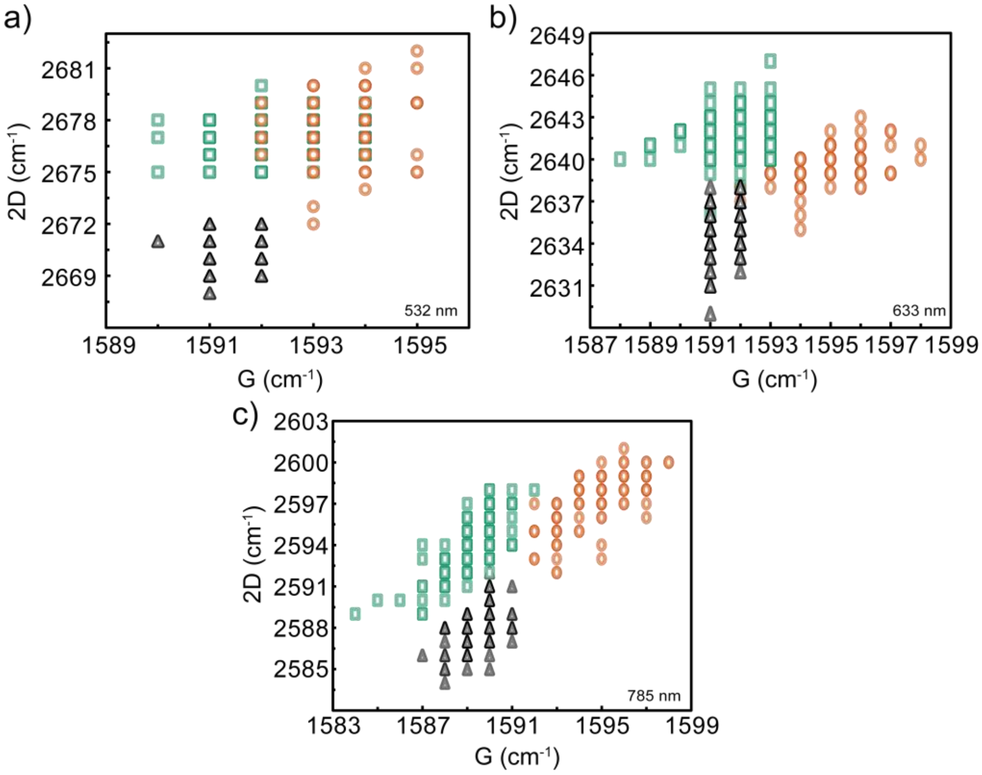

Supplementary Figure 9. **2D plot of Raman shifts.** Plots of the Raman shift of 2D band vs G band for 100 different spectra (λ_{exc} = 532 nm (a), 633 nm (b) and 785 nm (c)) of open SWCNTs (black triangles), SCO1@SWCNTs (green squares) and SCO2@SWCNTs (orange circles), data points are shaded to indicate the frequency of occurrence.

Supplementary Note 11. ATR-IR spectra

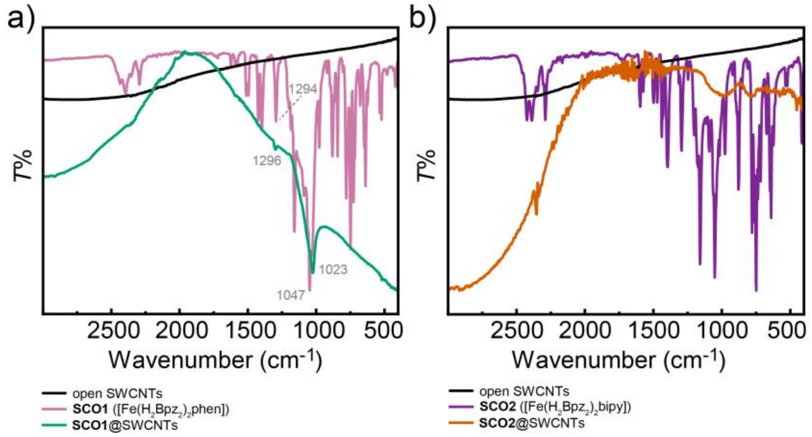

Supplementary Figure 10 **ATR-IR spectra.** a) ATR-IR spectra of open SWCNTs (black), SCO1@SWCNTs (green) and SCO1 (pink). b) ATR-IR spectra of open SWCNTs (black), SCO2@SWCNTs (orange) and SCO2 (purple).

Supplementary Supplementary Figure **10** shows the ATR-IR spectra of the two different complexes, encapsulated samples and pristine open SWCNTs. The pristine sample does not present any peak in IR,

while SCO1@SWCNTs presents two new peaks at 1296 and 1023 cm^{-1}. DFT/BLYP(mod)[2] calculations performed in the initial compound SCO1 show that the bands at 1294 and 1047 cm^{-1} -the ones that better match those of the encapsulated sample- correspond to the vibrations of the phenanthroline ligand. As in the case of Raman spectroscopy, confinement in the inner cavity of the SWCNTs restricts the vibration of compound SCO1 bonds. Frequency calculations in the final hybrid are currently being performed.

Supplementary Note 12. HAADF-STEM of a bundle of SCO1@SWCNTs

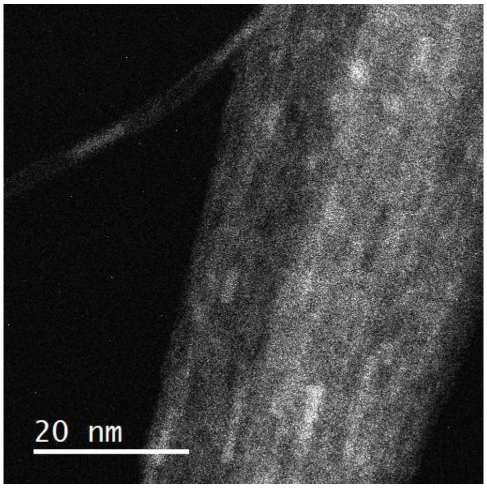

***Supplementary Figure 11*. HAADF-TEM of a bundle of SCO1@SWCNT.** a) HAADF-STEM image of a SCO1@SWCNTs bundle.

Supplementary Note 13. HAADF-STEM and EELS of pristine open SWCNTs

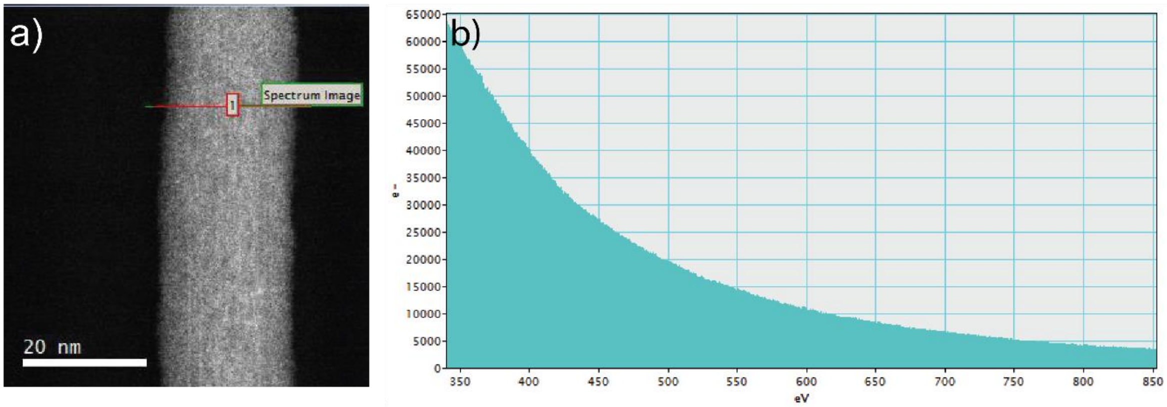

***Supplementary Figure 12*. EELS on pristine open SWCNTs.** a) HAADF-STEM image of a pristine open SWCNTs bundle. b) Average electron energy loss spectra (EELS) across de line selected in red in figure a).

Supplementary Note 14. Additional electron transport measurements and AFM images of devices

Supplementary Figure 13 shows the current-voltage (*IV*) characteristics measured at room temperature before and after deposition of a SCO2@SWCNT hybrid by dielectrophoresis. The current before dielectrophoresis (red curve) remains in the 10^{-12} A range. This is the noise level of our electronics and indicates that the gap between the electrodes is empty. In contrast, after dielectrophoresis, the current levels jump to the microampere range with a characteristic s-shape, typical of one or a few carbon nanotubes presenting Schottky barriers at the interface with the Au electrodes.

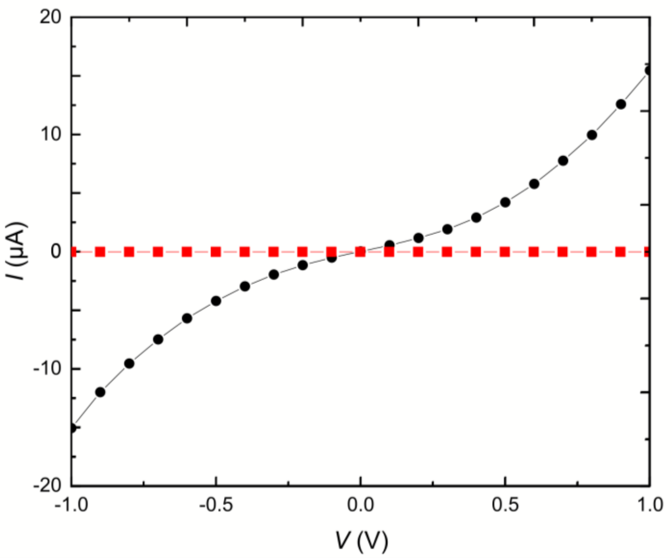

Supplementary Figure 13. **Trapping SWCNTs.** Current-voltage *IV* characteristic measured in a device before (red) and after (black) dielectrophoresis of a SCO2@SWCNT mixed-dimensional hybrid. The current level before dielectrophoresis points to an empty gap, whereas the current level and s-shape characteristic after dielectrophoresis is typical of one or a few carbon nanotubes presenting Schottky barriers at the interface with the Au electrodes.

Supplementary Figure 14 shows the temperature dependent current measured on the two samples presented in the manuscript together with four additional samples. All samples show the same thermally induced hysteresis in the conductance. The green lines are fits to the HS and LS curves using the Arrhenius law described in the main manuscript. The fitting parameters are summarized in Supplementary Table 2. In most of the cases, the HS to LS transition is associated with a drop in the activation energy *U* and the pre-exponential factor I_0.

Sample	*U*(HS) K	*U*(LS) K	I_0(HS) µA	I_0(LS) µA	I_i(HS) µA	I_i(LS) µA
1	1816(45)	891(15)	1770(29)	58(4)	11.73(0.01)	11.62(0.01)
2	4841(2271)	288(29)	4(32)	0.27(0.02)	0.45(0.01)	0.41(0.01)
3	1123(373)	329(23)	101(101)	3.8(0.4)	16.4(0.4)	16.68(0.02)
4	2528(227)	5400(420)	$16x10^3$	2(5)	13.48(0.01)	13.43(0.01)
5	560(83)	25(70)	2.9(0.8)	3(8)	2.70(0.03)	$2.34x10^{-6}$
6	1251(38)	1962(28)	0.49(0.01)	118.0(0.1)	$13.6x10^{-3}$	$14.0x10^{-3}$

Supplementary Table 2. **Arrhenius fit parameters.** Fitting parameters obtained for the curves presented in Figure 14.

The curves present an overall similar shape and T_{HC} and T_{LC} transition temperatures. Some of the peculiarities we observe are that the T_{HC} transition (from HS to LS) is barely visible in sample 2 whereas it cannot be observed in sample 6. This is due to the relative value of T_{HC} with respect to activation energy and exponential pre-factor from the Arrhenius fit, as explain in the main text. The closer the T_{HC} to the crossing between HS and LS conductance curves the more difficult to observe the HS to LS transition. It is also interesting to observe how in sample 3 there appear to be three distinct jumps from HC (red curve) to LC (blue curve). This probably indicates that different segments or clusters of the encapsulated SCO are switching from LS to HS at slightly different temperatures. Finally, sample 4 seems shifted to slightly lower temperatures close to bulk values.

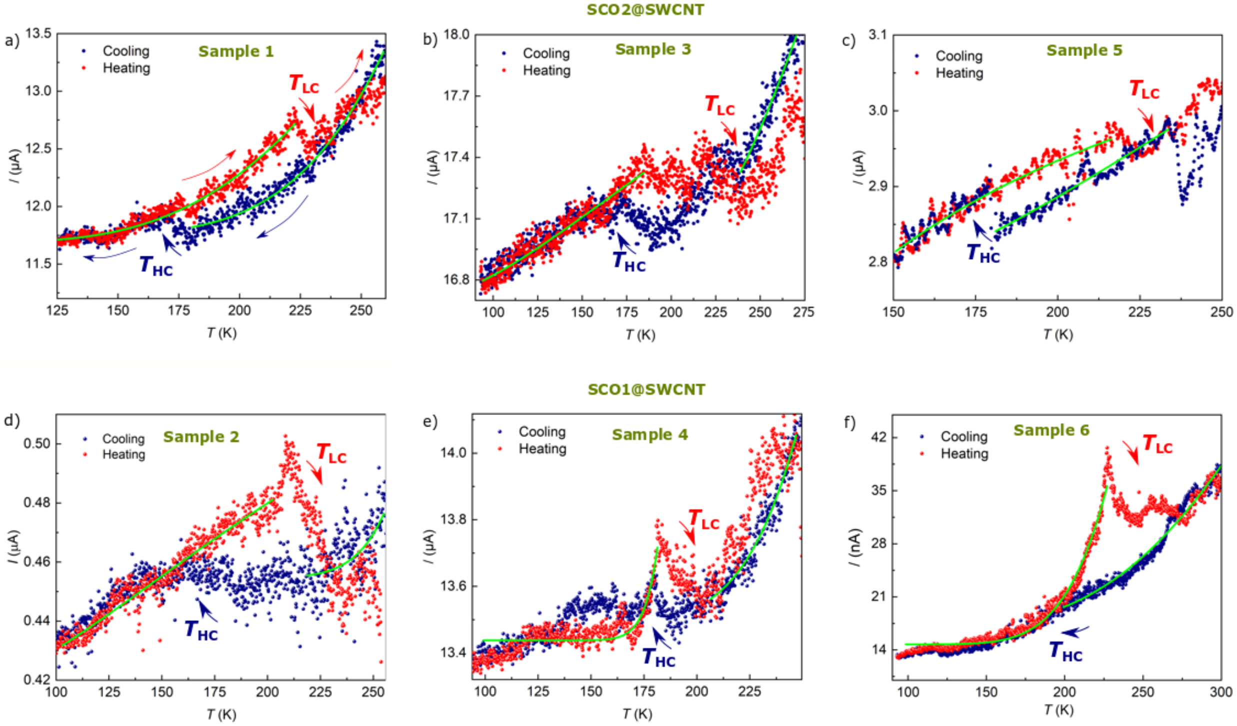

Supplementary Figure 14. **Additional samples.** Current I measured at a fixed bias $V = 1$ V as a function of the temperature in different SCO2@SWCNT (samples 1, 3 and 5) and SCO1@SWCNT (sample 2, 4 and 6) devices. The samples are initially cooled down to 90 K (blue curve) and subsequently heated up to room temperature (red curve). The conductance switches between a low-conductance LC and a high-conductance HC state describing a thermal hysteresis. The solid green lines are fits to the HC and LC states with an Arrhenius model for thermally activated transport.

It is interesting to note that although the resulting thermal cycles are roughly similar in all samples, there are a few differences between SCO1@SWCNT and SCO2@SWCNT hybrids. SCO2 gives rise to more elongated and flat hysteresis with well-defined T_{HC} and T_{LC} transitions. In contrast, SCO1 shows more asymmetric hysteresis loops with large and sharp T_{LC} jumps and barely visible T_{HC} transitions. The number of measurements does not allow thought to extract any solid conclusion in this respect. This will be studied in subsequent works.

We have also checked the stability and reproducibility of the hysteresis loop in sample 6. Supplementary Figure 15 shows the temperature dependence of the current measured in three consecutive thermal cycles. The key parameters of the hysteresis in the current are roughly stable and reproducible.

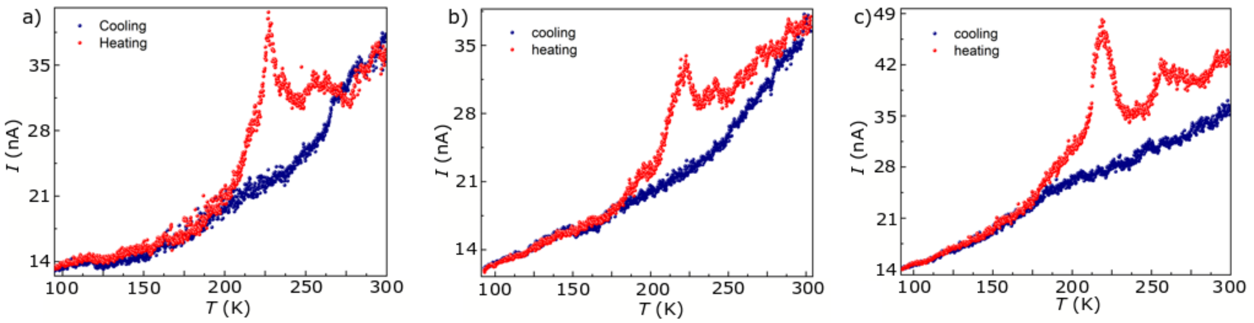

Supplementary Figure 15. **Reproducibility of the hysteresis.** Three consecutive thermal cycles measured on sample 6. The hysteresis is roughly preserved.

Supplementary Figure 16 shows the current measured as a function of the temperature in a test sample where the SCO molecules have been drop-casted onto pristine nanotubes already pre-aligned in a device. The devices are thereafter thoroughly cleaned with isopropanol and annealed in vacuum as the devices prepared in the manuscript. No hysteresis loop or transitions to different conductance levels are observed in the measurements.

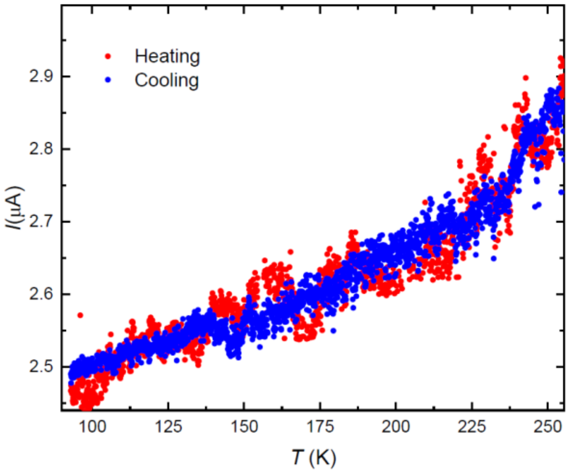

Supplementary Figure 16. **Supramolecular control sample.** Current I measured as a function of the temperature at a constant *V* = 1V in a test sample where the molecules have been deposited on supra and thereafter thoroughly washed and annealed. No sign of the thermal hysteresis or switch between metastable states are observed.

Supplementary Figure 17 shows an AFM image of a device containing at least one SWCNT bridging the gap between the Au electrodes in a nanodevice. The right part of the carbon nanotube is suspended from the electrode whereas the left part of the nanotube is resting on the substrate. The height of the tube observed in the profiles seems to indicate a single SWCNT.

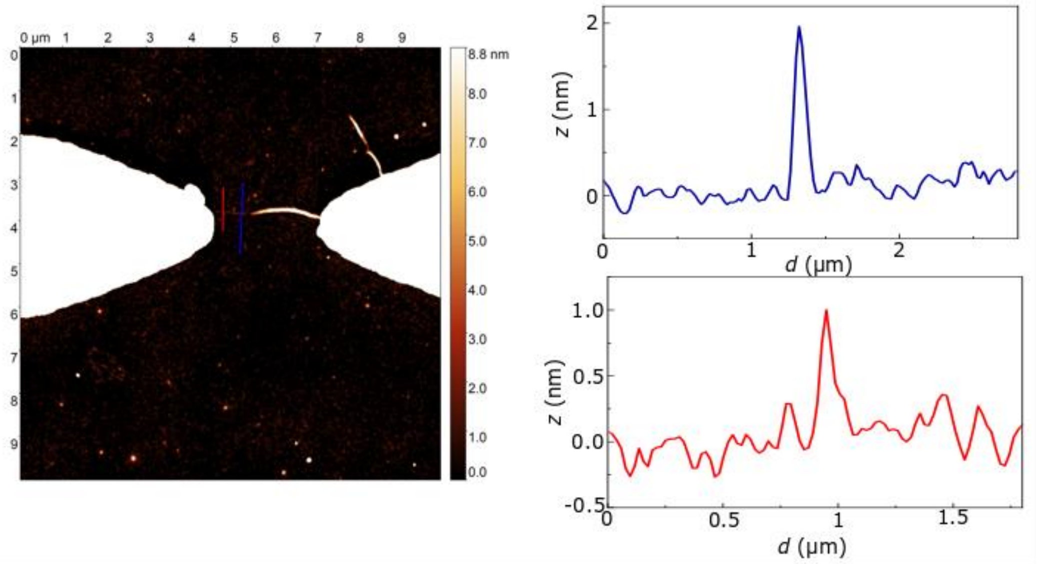

***Supplementary Figure 17*. AFM image of a SWCNT device.** AFM image and selected profiles of a nano-device containing a SWCNT.

Supplementary Note 15. Computational details

The adsorption of a single molecule on the single-walled carbon nanotube (16,8) has been studied within periodic density functional theory (DFT) using the VASP (Vienna ab initio simulation package) code[3, 4, 5, 6] employing the generalized gradient approximation (GGA) with the revised Perdew-Burke-Ernzerhof functional (rPBE)[7] and projector-augmented wave (PAW) potentials.[8, 9] rPBE functional has been proven to provide a good LS-HS balance (much better than other GGA functionals such as PBE) for well-known SCO complexes containing Fe(II) and Fe(III).[10] Valence electrons are described using a plane-wave basis set with a cutoff of 500 eV and the Γ-point of the Brillouin zone is used.[11] The optimized lattice parameters for the nanotube are a=b=16.75 Å and c=11.35 Å. Hence, the diameter of the nanotube once optimized is of 1.675 nm.

To study the encapsulation, we have used a 1x1x2 supercell with 37Å of vacuum between nanotubes (54 Å x 54 Å x 22.7 Å) containing 448 carbon atoms, and a single Fe SCO complex. Electronic relaxation has been performed until the change in the total energy between two consecutive steps is smaller than 10^{-6} eV and the ionic relaxation has been performed until the Hellmann-Feynman forces were lower than 0.025 eV/Å. As we are interested in the different magnetic solutions, the NUPDOWN option is used, which forces the difference between number of electrons in up and down spin channels, N_α-N_β, to be equal to 0 (LS) or 4 (HS). Several starting geometries for geometry optimizations have been tested. Interactions energies between the encapsulated SCO2 complex and the nanotube, E_{int}, were calculated as $E_{int}=E_{molecule@SWCNT} - (E_{SWCNT}+E_{molecule})$. A negative E_{int} value means that the encapsulated molecule is more stable than the free molecule.

Finally, the density of states (DOS) has been obtained from a single-point calculation on the optimized geo1 structure for each spin state with 1x1x9 k points.

The impact of an external electric field on the relative stability of the HS and LS spin states are analyzed through single-point density functional theory (DFT)-based calculations on the isolated molecule, using Gaussian 09 code. The lowest-energy geometry adopted by the molecule for each state, with the same orientation as in geo1, is employed in all these calculations. The TPSSh functional[12, 13] is used with the

def2SVP basis set[14] for all the atoms in the molecule. This functional has been reported to exhibit high accuracy for first-row transition metal systems, including iron SCO complexes.[10, 15]
The total energy of the molecule on the LS and HS states is evaluated under the effect of an electric field applied along the x, y or z directions. The strength of the electric field is expressed in atomic units, where 1 arb. unit = 5.14 10^9 V/cm.

Supplementary References

1. Real, J. A., Muñoz, M. C., Faus, J., Solans, X. Spin Crossover in Novel Dihydrobis(1-pyrazolyl)borate [$H_2B(pz)_2$]-Containing Iron(II) Complexes. Synthesis, X-ray Structure, and Magnetic Properties of [FeL{$H_2B(pz)_2$}$_2$] (L = 1,10-Phenanthroline and 2,2'-Bipyridine). *Inorg. Chem.* **36**, 3008-3013 (1997).
2. Reiher, M., Salomon, O., Hess, B. Reparameterization of Hybrid Functionals Based on Energy Differences of States of Different Multiplicity. *Theor. Chem. Acc.* **107**, 48-55 (2001).
3. Kresse, G. Ab initio molecular dynamics for liquid metals. *Phys. Rev. B* **47**, 4 (1993).
4. Kresse, G., Hafner, J. Ab Initio Molecular-Dynamics Simulation of the Liquid-Metal-Amorphous Semiconductor Transition in Germanium. *Phys. Rev. B* **49**, 14251-14269 (1994).
5. Kresse, G., Furthmüller, J. Efficiency of ab-initio total energy calculations for metals and semiconductors using a plane-wave basis set. *Comput. Mater. Sci.* **6**, 15-50 (1996).
6. Kresse, G., Furthmüller, J. Efficient iterative schemes for ab initio total-energy calculations using a plane-wave basis set. *Phys. Rev. B* **54**, 11169-11186 (1996).
7. Hammer, B., Hansen, L. B., Nørskov, J. K. Improved adsorption energetics within density-functional theory using revised Perdew-Burke-Ernzerhof functionals. *Phys. Rev. B* **59**, 7413-7421 (1999).
8. Blöchl, P. E. Projector augmented-wave method. *Phys. Rev. B* **50**, 17953-17979 (1994).
9. Kresse, G., Joubert, D. From ultrasoft pseudopotentials to the projector augmented-wave method. *Phys. Rev. B* **59**, 1758-1775 (1999).
10. Siig, O. S., Kepp, K. P. Iron(II) and Iron(III) Spin Crossover: Toward an Optimal Density Functional. *J. Phys. Chem. A* **122**, 4208-4217 (2018).
11. Monkhorst, H. J., Pack, J. D. Special points for Brillouin-zone integrations. *Phys. Rev. B* **13**, 5188-5192 (1976).
12. Tao, J., Perdew, J. P., Staroverov, V. N., Scuseria, G. E. Climbing the Density Functional Ladder: Nonempirical Meta-Generalized Gradient Approximation Designed for Molecules and Solids. *Phys. Rev. Lett.* **91**, 146401 (2003).
13. Staroverov, V. N., Scuseria, G. E., Tao, J., Perdew, J. P. Comparative assessment of a new nonempirical density functional: Molecules and hydrogen-bonded complexes. *J. Chem.Phys.* **119**, 12129-12137 (2003).
14. Weigend, F. Accurate Coulomb-fitting basis sets for H to Rn. *Phys. Chem. Chem. Phys.* **8**, 1057-1065 (2006).
15. Kepp, K. P. Theoretical Study of Spin Crossover in 30 Iron Complexes. *Inorg. Chem.* **55**, 2717-2727 (2016).
